# Don't Take It 'Lytely': A Case of Acute Tetany

**DOI:** 10.7759/cureus.5845

**Published:** 2019-10-05

**Authors:** McKenna M Johnson, Satya Patel, Jason Williams

**Affiliations:** 1 Internal Medicine, David Geffen School of Medicine at University of California Los Angeles, Los Angeles, USA; 2 Internal Medicine, University of California Los Angeles, Los Angeles, USA; 3 Internal Medicine, Louis Stokes Cleveland Veterans Affairs Medical Center, Cleveland, USA

**Keywords:** tetany, hypocalcemia, respiratory alkalosis, crohns disease, hypokalemia, rhabdomyolysis, hypomagnesemia

## Abstract

The most common causes of tetany are hypocalcemia, hypomagnesemia, hypokalemia, and alkalosis. Most case reports of tetany in the literature include some combination of the above metabolic derangements leading to non-life-threatening symptoms. We present a unique case of severe life-threatening tetany in a 38-year-old female with a history of Crohn’s disease. She was previously dependent on total parenteral nutrition (TPN) but discontinued TPN two weeks prior to presentation due to the improvement of her Crohn’s symptoms with a new medication regimen. We propose that malabsorption led to multiple electrolyte abnormalities, resulting in acute tetany that subsequently caused rhabdomyolysis. This case reviews the most common causes of acute tetany and highlights the interaction between electrolytes implicated in both tetany and rhabdomyolysis. It also emphasizes the importance of considering tetany as a diagnosis in a patient with unstable vital signs and diffuse muscle spasms.

## Introduction

Tetany is a condition in which an abnormal serum electrolyte concentration, such as hypocalcemia, hypomagnesemia, hypokalemia, or alkalosis, leads to neuromuscular irritability. Often, the etiology is not due to a single cause but rather a combination of electrolyte derangements. Several cases in the literature describe the development of tetany in young females with malabsorption due to inflammatory bowel disease, celiac disease, or bulimia, who are found to have multiple electrolyte abnormalities [1-3]. The majority of published cases of tetany describe non-life-threatening symptoms. We present a case of acute life-threatening tetany in a 38-year-old female with chronic malabsorption due to Crohn’s disease.

## Case presentation

A 38-year-old female with a history of treatment-refractory Crohn’s disease with prior ileocolectomy presented to the emergency department with acute onset of severe muscle cramping and spastic paralysis. She failed numerous therapies for Crohn’s disease in the past, including mesalamine, 6-mercaptopurine, infliximab, adalimumab, and vedolizumab. As a result, she developed chronic malabsorption and intermittently required total parenteral nutrition (TPN). Her primary indication for TPN was refractory hypokalemia. However, due to a new treatment regimen with ustekinumab initiated two months prior, her symptoms improved, and she stopped TPN therapy two weeks prior to presentation. She reported a normal appetite and good oral intake during this time period. She endorsed mild diarrhea, which was consistent with her baseline after the ileocolectomy. Of note, she did not have any laboratory tests since the cessation of TPN. 

**Figure 1:**
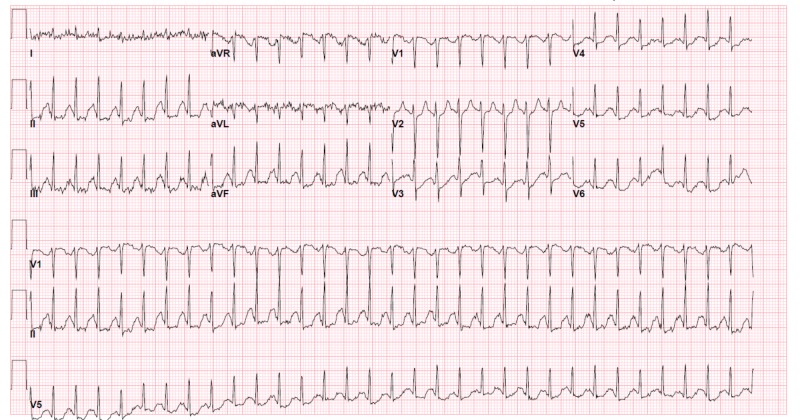
EKG during initial presentation in emergency department

**Figure 2:**
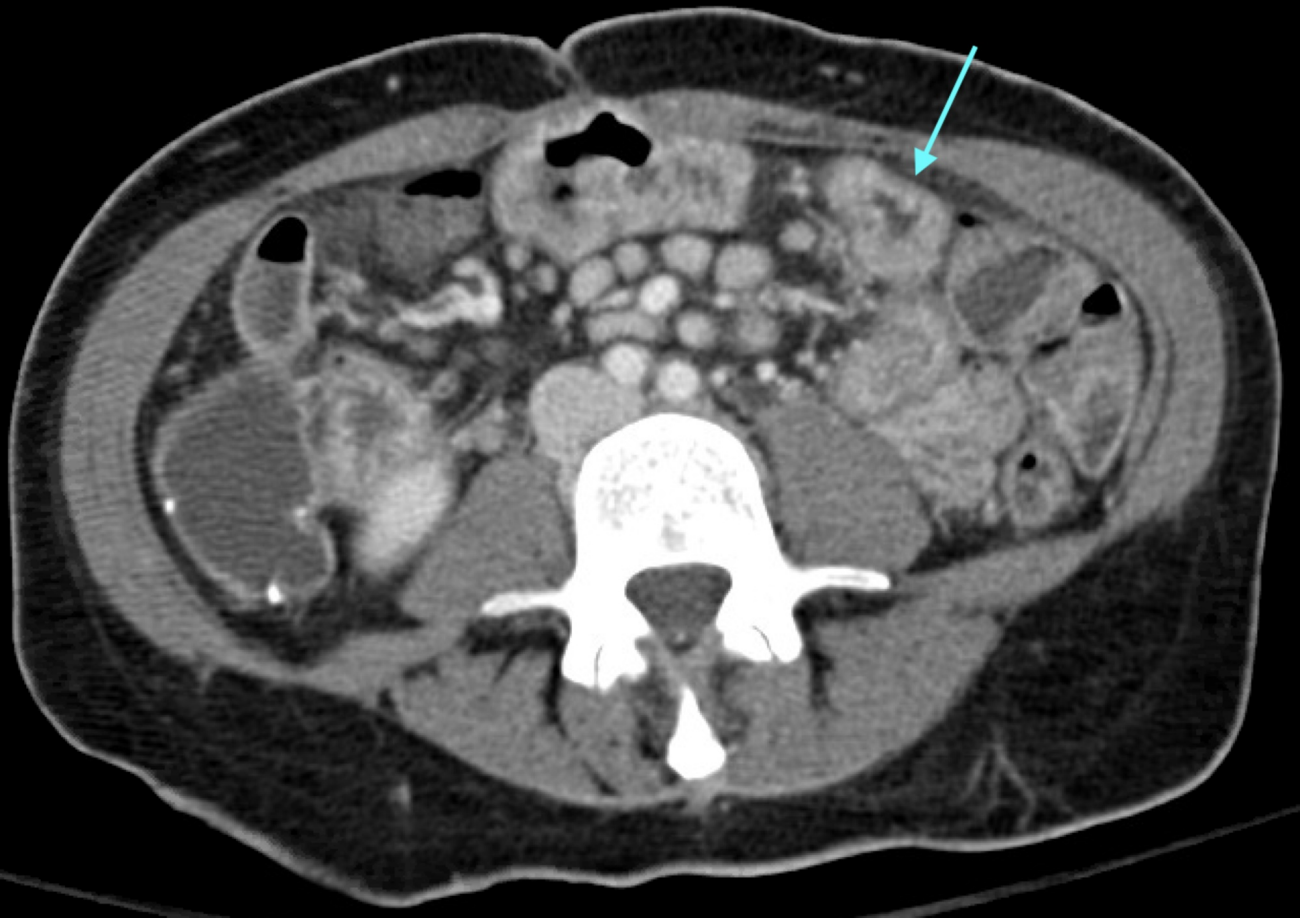
CT abdomen and pelvis with IV contrast The blue arrow points to the region of small bowel wall edema.

A few hours before presenting to the emergency department, she was setting up tables for a work conference when she noticed sudden-onset perioral numbness and tingling of her hands. Within minutes, she developed progressive and severe muscle cramping. She described finger extension, wrist flexion, arm flexion against her chest, leg extension, rotational torticollis to the left, and shortness of breath. On arrival to the emergency department, her vital signs were as follows: temperature 40.7°C, heart rate 192 beats/minute, blood pressure 102/60 mmHg, respiratory rate 40 breaths/minute, and normal oxygen saturation on room air. Physical exam revealed an alert, diaphoretic female in acute distress with diffuse abdominal tenderness and diffuse muscle spasm. Her initial labs included the following: potassium 4.0 mEq/L, lactate 87 mg/dL (9.65 mmol/L), creatinine kinase (CK) 150 U/L, and a venous blood gas with pH of 7.46 and pCO2 of 24 mmHg. Additional laboratory values can be seen in Table 1. Serum calcium and magnesium were not initially tested. Her initial electrocardiogram (EKG) can be seen in Figure 1. CT head without contrast showed no sign of intracranial hemorrhage. Due to her acute and severe presentation, she was empirically treated for sepsis with intravenous (IV) fluids, antibiotics, and antifungals. IV fluids administered included 1L normal saline and 1.5L lactated ringers. CT abdomen and pelvis with IV contrast was notable for hyperenhancement and bowel wall edema of the distal small bowel, most likely due to inflammatory bowel disease or infectious enteritis (Figure 2). Within three hours of arrival, her symptoms resolved and lactate normalized. However, her serum CK increased significantly, peaking at 12,380 U/L. Six hours after presentation, her labs were notable for the following: potassium 2.4 mEq/L, calcium 6.3 mg/dL (corrected calcium 7.8 mg/dL), and magnesium 0.9 mg/dL. Aggressive electrolyte repletion was initiated. Her clinical exam normalized the following morning, and IV antibiotics and antifungals were discontinued. Gastroenterology was consulted to address her severe malabsorption and recommended an additional dose of ustekinumab along with the resumption of TPN.

**Table 1:** Laboratory values during the first two days of the hospital course Normal range: calcium, 8.6-10.3 mg/dL; magnesium, 1.4-1.9 mEq/L; potassium, 3.6-5.3 mmol/L; phosphorous, 2.3-4.4 mg/dL Abbreviations: BUN, blood urea nitrogen; PTH, parathyroid hormone

Laboratory Values		Day 0		Day 1						Day 2
		9:30 PM	12:00 AM	12:45 AM	4:00 AM	8:00 AM	10:00 AM	6:00 PM	10:00 PM	5:00 PM
Sodium (mmol/L)		140			144		143	146		145
Potassium (mmol/L)		4			2.4		3	3.1		3.2
Chloride (mmol/L)		100			105		107	110		109
Bicarbonate (mmol/L)		18			23		22	19		20
BUN (mg/dL)		8			7		14	4		16
Creatinine (mg/dL)		0.8			0.86		0.76	0.7		0.63
Lactate (mg/dL)		87	13	9						
Creatinine Kinase (U/L)		150		5077	9066		12380	12205		8555
Calcium (mg/dL)					6.3		7.5	7.4		7
Magnesium (mEq/L)					0.9		2.3			2.7
Phosphorous (mg/dL)								1.5		2.9
Albumin (g/dL)					2.1			2.4		
Ionized Calcium, Corrected (mmol/L)								0.97		
PTH, intact (pg/ml)								32		
Vitamin D, 25-OH (ng/mL)								16		
Venous Blood Gas (pH/pCO2)		7.46/24								

## Discussion

Due to the acuity of her presentation and the combination of clinical symptoms, the differential diagnosis included sepsis, seizure, malignant hyperthermia, and serotonin syndrome. However, none of these diagnoses could entirely account for her presentation. Although sepsis was considered due to her fever, tachycardia, and prior peripherally inserted central catheter (PICC), she had no obvious source, and finalized cultures revealed no evidence of infection. Seizure was considered due to her widespread muscle contractions and unusual posturing, yet her mental status was unaffected, and she had no prior history of seizures. Her hyperthermia raised suspicion for malignant hyperthermia or serotonin syndrome, but she had no exposure to neuroleptic agents or serotoninergic medications. We believe that her symptoms can be explained by acute tetany followed by acute rhabdomyolysis. Tetany likely caused her diffuse muscle spasms, leading to the development of rhabdomyolysis, which caused fevers and tachycardia. Additionally, the marked level of tachycardia at 192 bpm was most likely due to the development of arrhythmia related to electrolyte abnormalities.

Tetany is a condition caused by peripheral nerve irritability, which leads to repetitive, high-frequency discharges. The most common signs are perioral numbness, acral paresthesias, and muscle spasms. Chovstek and Trousseau signs are two classic physical exam signs which demonstrate nerve hyperreactivity and suggest tetany. Chovstek sign is elicited by tapping the facial nerve anterior to the ear, which causes contraction of ipsilateral facial muscles, such as twitching of the lip. Trousseau sign is the development of carpopedal spasm with inflation of blood pressure cuff above systolic blood pressure for three minutes [4]. Carpopedal spasm is a specific form of involuntary cramping in the hands and feet nearly synonymous with tetany. In the hands, patients develop involuntary finger extension, wrist flexion, thumb abduction, and metacarpophalangeal joint flexion. A similar pattern of cramping occurs in the feet. In some cases of tetany, muscle cramps may progress to generalized muscle contractions, as seen in our patient. Additionally, autonomic effects can also be seen, such as diaphoresis, bronchospasm, and biliary colic [5]. When using physical exam signs to aid in the diagnosis of tetany, it is important to note that the Trousseau sign is reasonably sensitive and specific for hypocalcemic tetany, but Chovstek sign is neither sensitive nor specific. For example, in patients with hypocalcemic tetany, Trousseau sign is present in 94% of patients with hypocalcemia and only 1% of patients with normal calcium levels. However, Chovstek sign is present in 10% of patients with normal calcium levels and absent in about 30% of patients with hypocalcemia [6].

Hypocalcemia is the most well-known cause of tetany, defined as serum calcium level below 7.5 mg/dL [5]. It can also cause cardiac conduction disturbances, decreased myocardial contractility, seizure, and psychiatric manifestations [5]. In this patient, her initial EKG showed supraventricular tachycardia, which may have been caused by hypocalcemia (Figure 1). The swift reversal of her tetany and stabilization of clinical conditions were likely due to the use of calcium-containing Lactated Ringer’s solution during initial resuscitation. 

Respiratory alkalosis and hypokalemia, both seen in our patient, can also contribute to tetany. Respiratory alkalosis causes dissociation of bound hydrogen ions, which bind to free serum calcium, causing hypocalcemia. Alkalosis can also independently cause nerve irritability [7]. Furthermore, reports of tetany associated with hypokalemia in the absence of other metabolic derangements have also been reported, suggesting that hypokalemia can independently cause tetany [8,9].

Hypomagnesemia is another important cause of tetany, as magnesium affects the metabolism of both calcium and potassium. Low magnesium levels may reduce the threshold for hypocalcemia to cause tetany. Although acute hypocalcemic tetany is not usually seen unless corrected serum calcium levels are below 7.5 mg/dL or serum ionized calcium levels are below 1.1 mmol/L, the condition can be seen at higher calcium levels if serum magnesium levels are low [5,10]. Hypomagnesemia may have also contributed to both tetany and rhabdomyolysis, in our patient by affecting potassium metabolism. Low magnesium levels cause increased renal potassium wasting, which potentiates pre-existing hypokalemia. Until hypomagnesemia has been corrected, potassium repletion will not effectively correct hypokalemia [11,12]. 

**Figure 3:**
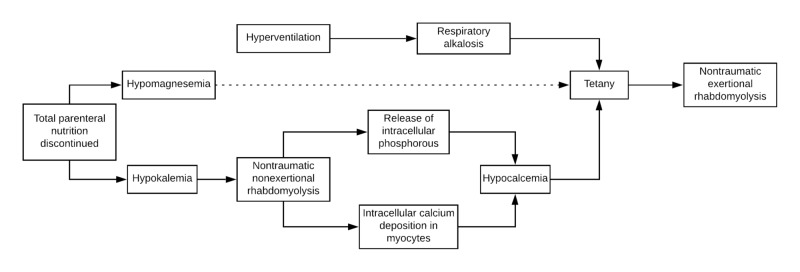
Theory of clinical events if triggered by hypokalemia-induced rhabdomyolysis

In this patient, tetany caused diffuse muscle contractions, which precipitated acute rhabdomyolysis. The potential causes of rhabdomyolysis can be broken down into one of three categories: traumatic, nontraumatic exertional, or nontraumatic nonexertional [13]. Electrolyte disorders, particularly hypokalemia and hypophosphatemia, can cause nontraumatic nonexertional rhabdomyolysis [14,15]. In this patient, the development of generalized muscle contractions caused nontraumatic exertional rhabdomyolysis. In addition, due to the presence of persistent hypokalemia after discontinuing TPN, it is possible that she also developed preceding nontraumatic nonexertional rhabdomyolysis prior to presenting with tetany. If so, the release of intracellular phosphorous from damaged myocytes could have contributed to acute hypocalcemic tetany by binding free serum calcium (Figure 3) [16]. 

This patient’s alarming clinical picture was ultimately caused by the development of multiple concurrent electrolyte derangements leading to life-threatening tetany. In the combined setting of hypocalcemia, hypomagnesemia, and respiratory alkalosis, our patient developed acute tetany with diffuse muscle contractions. The development of diffuse muscle contractions then precipitated a more severe nontraumatic exertional rhabdomyolysis. 

## Conclusions

In the setting of severe malabsorption, metabolic derangements can have life-threatening consequences. In this case, undetected ongoing malabsorption of potassium, magnesium, and calcium caused a presentation of acute, severe tetany. The vignette highlights the importance of detecting and correcting electrolyte abnormalities as they can ultimately lead to hemodynamic instability. 
